# Supplementary material for: Integrating noncommunicable disease care in a public primary health care facility in North Lebanon: A qualitative study of implementation in a humanitarian crisis
Source: PLOS Glob Public Health. 2026 Apr 1;6(4):e0005518. doi: 10.1371/journal.pgph.0005518 (PMC13042628; doi:10.1371/journal.pgph.0005518)
Supplement: S2 File — (DOCX) [file pgph.0005518.s002.docx]

***Supplementary Material 2: Interview Topic Guides (Patients and Caregivers)***

**Topic Guide for Patients**

1. **Demographics:**

- Age
- Gender
- Area of Residence

1. **Clinical information**

- What type of NCD(s) do you have? (Type 1 Diabetes, Type 2 Diabetes, Hypertension, Combination of these)
- When and how were you diagnosed with this NCD? What symptoms do you experience, and does it influence your daily life? What are your difficulties in managing for the illness?

**3. Experience at CAJA Site**

- Can you tell me why and how you started attending this clinic? Why this clinic and not another? How often do you come to the clinic, and why? Is it easy for you to access NCD-related care at this center? How do you come to the clinic, how long does the journey take you?
- Have you faced any challenges or obstacles in receiving NCD services here? please tell us about your experience.
- Are there any barriers that make it difficult for you to follow your NCD treatment plan? What could be done to address these barriers?
- How has the CAJA clinic influenced your health? Are the services provided meeting your needs? Have you required special medicines or assistive technologies to help with your daily function- have you been able to receive this? Has this process been easy or difficult- please tell us about your experience.

**4. Integration of services**

- In the last 6 months, have you noticed the integration of medical, psychosocial and physio services into the primary care you receive? How do you feel about the integration of these services?
- Can you describe your experience in the triage room when you first arrive at the clinic? Did you find the process efficient and helpful in determining your care needs? Can you share any positive or negative experiences with healthcare providers?
- What have you observed in your interactions with the health professionals and staff in CAJA? Have you observed any changes in the last few months this year? What changed? When? What are your thoughts about the changes? Do these changes (if any) affect/help how you manage your illness?

**5. Quality of NCD care**

How would you rate the quality of care you have received for your NCD(s) at this center? Can you share any specific experiences with services related to NCDs?

**6. Communication and Education:**

- Do healthcare providers communicate effectively about your NCD(s) and treatment plans? Do they give enough time for your consultation and take time to answer your questions?
- Have you received adequate education and information regarding your NCD(s) at this center? Have you benefited from any community or peer support programs related to NCDs here? Can you share your experience?

1. **Suggestions for Improvement:**

- What changes or improvements would you recommend making NCD services more effective and patient-friendly at this center?
- Do you have any ideas on how to enhance patient education and awareness about NCDs within the center?
- Has the clinic asked for your feedback and suggestions, to better cope with your condition?
- Do you have any suggestions for the clinic to help you manage your health and attend the clinic easier?

**Topic Guide for caregiver**

1. **Caregiver Demographics:**

- Age (patient and caregiver),
- Gender (patient and caregiver),
- Relationship to the person receiving care,
- Length of time as a caregiver

1. **Experience with the Person You Care For:**

- What are the primary responsibilities or tasks you handle as a caregiver? How many hours a day or week do you typically spend caregiving? Are there specific challenges or difficulties you face in your caregiving role?
- Can you tell us about the person you are caring for and his/her NCD?
- How was his/her NCD diagnosed, and what symptoms do he/she experience? How does it influence their daily life? What difficulties have you encountered in managing their illness?
- How has the CAJA clinic and caregiving influenced your own health and well-being? Have you required any special assistance or support in your caregiving role?

1. **Experience at CAJA Site**

- How and why did you and the person you care for start attending this clinic? Can you share your reasons for choosing this clinic over others? How often do you come to the clinic, and why? Is it easy for you to access NCD-related care at this center? How do you come to the clinic, and how long does the journey take you?
- Have you encountered any difficulties in accessing or receiving care for the person you are caring for at this clinic? Are there any barriers that make it challenging to follow the NCD treatment plan for the person you care for?

**4. Integration of services at CAJA site**

- Inquire if they have noticed the integration of NCD services into the primary care at the CAJA clinic in the last 6 months: How do you feel about the integration of NCD services into CAJA's PHCs?
- Can you describe your experience in the triage room? Did you find the process efficient and helpful in determining care needs? Can you share any positive or negative experiences with healthcare providers in the triage room?
- What have you observed in your interactions with the healthcare professionals and staff at CAJA? Have you observed any changes in the clinic's services recently? How do these changes affect how you and the person you care for manage the illness?

1. **Quality of care**

How would you rate the quality of care provided for the NCD(s) at this center? Can you share any specific experiences with services related to NCDs?

1. **Communication and Education:**

- Do healthcare providers effectively communicate about the NCD(s) and treatment plans?
- Have you received adequate education and information regarding the NCD(s) at this center?
- Have you benefited from any community or peer support programs related to NCDs here?

**7. Suggestions for Improvement:**

- What changes or improvements would you recommend making NCD services more effective and caregiver-friendly at this center?
- Do you have any ideas on how to enhance caregiver education and support within the center?
- Have you been asked for feedback and suggestions by the clinic to better cope with caregiving needs?
- Do you have any suggestions for the clinic to help caregivers manage their health and caregiving responsibilities more effectively?
