# Supplementary material for: Integrating noncommunicable disease care in a public primary health care facility in North Lebanon: A qualitative study of implementation in a humanitarian crisis
Source: PLOS Glob Public Health. 2026 Apr 1;6(4):e0005518. doi: 10.1371/journal.pgph.0005518 (PMC13042628; doi:10.1371/journal.pgph.0005518)
Supplement: S3 File — (DOCX) [file pgph.0005518.s003.docx]

***Supplementary Material 3: Axial Codes And Themes Reported Against REAIM-PRISM Implementation Research Domains***

| **Themes and Axial Codes** | ***Example quotes*** |  |
| --- | --- | --- |
| ***Adoption*** | | |
| Clear communication and collaboration process | *We were always in collaboration, step by step, to develop and carry out the process. They first informed us, and we agreed to provide integrated health care. We, of course, welcomed such a project (CAJA)* |  |
| Getting wider buy-in by leveraging influence of trusted and respected community leaders | *Having the director on your side is something that would really… I'm not really a pointing out to the power thing but having him convinced of the importance of this services at the PHC really helped us in terms of I'm having the staff available for this… (ICRC)* |  |
| Openness of all parties to collaborate and try new approaches | *The staff of CAJA very welcoming to new ideas (ICRC)* |  |
| ***Implementation*** | | |
| Role and process clarity | *The way things are going, you know your job, you are responsible for this and this. let's say now it's clear this is not my job and this is my job and it's make less, let's say conflict in in these things because sometimes someone is doing more or less (ICRC)* |  |
| Initial steep learning curve and resistance | *Honestly, in general, when an employee is told that he has more work, he starts to complain at first, but then, everything went well and easily. He thought it would be a difficult task. The process is easy, and it is well carried out. At the beginning, it was difficult, and everyone complained, saying that we would not be able to implement such a project as we would have more work to do. (CAJA)*  *In the beginning, the employees were surprised, and they needed time to train and follow the international standards (CAJA)* |  |
| Stepwise and frequent follow up supervision by ICRC | *The follow up you do with us. I mean, when we started, it was not 100% of my work, but when I noticed your follow-up, [he mentions the names of the ICRC team], you were telling us how to work, how to perform the diagnosis, and we should be more careful, [incomprehensible], or we should... Such details have developed us more. The main reason that helped us to implement this project is you (CAJA).* |  |
| ***Reach*** | |  |
| PHC for the family | *I noticed that many of our old patients for two years ago, are now bringing their families with them because they are benefiting. They found themselves in a better condition, they are receiving very good services, and they are being followed up. Hence, we have more of those cases (CAJA).* |  |
| Refusal of MHPSS | ***Stigma:*** *It's the old mentality that mental health is for the crazy people. (ICR)C; the ones who refuses, either because of the stigma or because they didn't want to come to the PHC on a weekly basis. So I don't think they know the value the true value of the service (ICRC)* |  |
|  | ***Lack of trust:*** *Because I do not trust such a thing (P6)* |  |
|  | ***Preference for faith-based coping****: Even though I had been taking the (psychotropic) medication for 32 years, I did not benefit from it. I benefited more from praying. When the person prays and seeks forgiveness from God, I notice that I do not need the antidepressant. Why should I take it when it has side effects, and it does not help me? (P4).* |  |
|  | ***Aversion to MHPSS medicine but open to talk therapy:*** *I might accept attending a session, like a session with you. However, I am afraid of taking a medication because whoever takes it will get used to it and become mad. Now I am facing a difficult situation, and I could get over it in one week, or one or two months, and hopefully, then, I will get back to my normal life. I am facing a difficult situation, and I do not like to take medication for now. (P6)* |  |
| Success in addressing some levels of cultural stigma and taboos. | *Regarding mental health when we started with the ICRC at the beginning, I told them that our environment might not be interested in this issue. Even in people’s culture, mental health is a taboo; and if someone suffers from an issue, they try to hide it, do not provide him with the right treatment, or treat him in a non-medical way. That exists in our culture. I told them that it might be difficult, but with time, experience, and mechanism, society has accepted it. I did not expect patients with psychological issues to come to the psychiatric clinic. Thank God, psychiatry has become a normal thing for people, and they have accepted it. (CAJA)* |  |
| Realising and acknowledging MHPSS benefit | *On my first visit, I thought that what happening to me was normal. But when I talked to her, I benefited a lot, and I learned a lot from her… Since I have started talking to her, I can understand more the situation and behave differently at home for sure. I feel more relaxed. I follow her advice. I feel more comfortable. (P1)* |  |
| Shortage or unavailable access to medicines, device or prosthesis supply | ***Medicine:*** *If the medication was not provided by the centre, I would not have bought it; I have access to services and get my medications every month from the centre. Had I been unable to get it from here, I could not have afforded it.; Half of the people in Akkar cannot afford medical care. The situation is bad (P3)*  *Nowadays, we are searching for a dispensary where we can find medications. Sometimes you go to the dispensary, they examine you, and you pay, but you do not find any medication (P2)* |  |
|  | ***Prosthetics and diabetic shoes:*** *Faced supply challenges for prosthetics and shoes last year- due to financial challenges, and organizational changes (ICRC)* |  |
|  | ***Test strips****: on my first visit to get the treatment here, they referred me to the endocrinologist. I visited her, [incomprehensible]. She asked me to do some tests, but I could not afford to buy the strips, so I stopped visiting her. She asked me to do the tests three times a day for a week, but I could not afford the... I have the device at home, but I could not afford the strips. I stopped visiting her clinic, and I came back to Doctor (PHC at CAJA) (P1)* |  |
| Transport challenges weekly physio or MHPSS | *the transportation challenges that would also be an important point to highlight because not all people are close to the PHC and they are, they are used to come to, to the PHC like as you know for the NCD's, it's not like a weekly follow up, but for the mental health sessions, you would ask them to come every week. (ICRC)* |  |
| ***Adaptations*** | | |
| Having flexible staff and processes | *flexibility to adapt and we have staff that are also very agile (ICRC)* |  |
| Having the authority and ability to get team consensus to jointly address a challenge or limitation | *Recognizing the need for more effective awareness sessions, I collaborated with the CAJA team to implement group sessions in the waiting room. This decision came after discussions with the social worker, who proposed organizing sessions for 3 to 4 patients with similar conditions during waiting times. I was surprised with the enthusiasm of the social worker for this change. She expressed her excitement to the proposed change, and she committed to initiating these sessions starting early next week, dedicating one day per week for this purpose. (ICRC)* |  |
| Exploring and pivoting to more efficient alternatives | *During this journey, we tried to open a laboratory. We launched the laboratory, but it turned out that if we worked with the main large laboratories, it would still be more affordable for the patient, so we resorted to the second solution. That was a way to alleviate because the laboratory and blood test costs are expensive for patients (CAJA)* |  |
| Team-based decision-making and experiment to solve challenges | *scheduling appointments- may not have been on same day as GP visit let’s say, and with different days- people think about the additional cost, time, energy of coming in only for PRP; so they tried to merge medicines pick up with PRP appointments (ICRC)* |  |
| ***Effectiveness*** | | |
| Staff observed changes in service users | *you can see their scores on the scales pre- and post-intervention, and you can see that there is always an improvement in terms of symptomatology (ICRC)* |  |
| Mindsets and perceptions of value (of integrated care) | ***Long-term saving:*** *If you do the correct approach you are saving, so for me I think it is fine to put some budget and to spend some money on the preventive approach. (ICRC)* |  |
|  | ***International standards****: this is an excellent move, not in my opinion, but in the opinion of patients themselves. This is a distinguished medical service based on European standards, or at least, unavailable standards in a remote area, like Akkar, which they always call the deprived remote area. We are not used to such standards (CAJA f)* |  |
|  | ***People centred care and health awareness****: The patient now knows about his case. I can say that it was like a dream to know that an employee called the patient and told him that he must come to get his medication, which is an unusual thing for the patient (CAJA f)* |  |
|  | ***Changing work mindsets:*** *They didn't have this mindset before-.I mean, why should we examine a diabetic patient…Awareness itself is important, even if there was no funds to donate anything or so, you created a new mindset for the people that are working outside ICRC. (ICRC)* |  |
| Healthy behaviour change | *Since I have cholesterol and triglycerides, he tells me to stop eating white bread completely. We apply his recommendations, and we are seeing good results. I stopped eating white bread. [Incomprehensible] [I now eat] home meals, beans, and vegetables. I am having better results. (P3)* |  |
| Service user satisfaction- relational quality | *I find this centre different than any other centre, in terms of the nurses, the supervisors and the doctors. They respect the patients. In other dispensaries, they do not respect the patients. (P8)*  *Honestly, I feel relieved after visiting him. I have never received a treatment like Doctor [Doctor's name]. I truly like consulting, in terms of his advice and his treatment. I feel comfortable with him. Some doctors only prescribe the medications, but the doctor discusses them with me. (P1)* |  |
| Meeting health needs | ***Holistic care:*** *you cannot focus only on the disease, because if (patient) is not feeling well, if he's depressed, he's not gonna be better. Even if he took the medication, he's not gonna improve very much if we didn't care for his foot, he might end up with amputation. He will be more sick. (ICRC)* |  |
|  | ***Non-target NCD related health needs that service users also reported:*** *osteoarthritis, aortic aneurysms, kidney stones, congenital hearing problems, insomnia, deep vein thrombosis* |  |
| Matching increase of reach and volume of service users with quality and timely service delivery | *I think it is important to take into consideration the patient load and the services given. when you're overloaded and they have a lot of patients and everyone, you have to look through if he needs a referral. And so it is important, I think, to see a lot of patients, but also it is important to reserve the quality of the service so to keep a balance between how many patients we are seeing, (ICRC).* |  |
| Unintended consequences | ***Comparison seen as unequal:*** *Sometimes service users talk to each other, and when one is offered diabetic shoes or prosthetics based on their risks and need, others will question why they were not offered the same (ICRC)* |  |
|  | **Wait time**: The only annoying issue is waiting for so long. Sometimes some patients come to the centre on an empty stomach to do blood tests, and they come from a remote area. (P2) |  |
| ***Maintenance & sustainability*** | | |
| Completion of implementation-as process ‘runs on its own’: the new normal | *It feels like it's running on its own. We are now at the best days of integration because all the components are there, we just lack the medication and the PHC, and we are working on it (ICRC).* |  |
| Community loyalty leads to low turnover | *So, this is your this is your communities, your neighbours, you’re your family, your town-so you have a certain loyalty, loyalty to them who you are helping. They (Local staff) are changing lives, and anyone who's working in this field and can make a difference. (ICRC)* |  |
| Positioning and equipping NGO as health system partner | *They become key partners for the ministry in the delivery of care and this region, and this is amazing. (ICRC)* |  |
| Linkage to MOPH programme | *the National Mental health programme will ensure that it's not only a pilot project anymore, it's gonna be a sustainable thing for all PHC's. (ICRC)* |  |
| Justifying ICRC cut of programme | *And also don't forget that in Lebanon, we're able to do this to cut to our programme because there are so many other organizations on the PHC side in countries. Continuity in CAJA was based on this research partnership and funding- without which, it would have been cut. (ICRC)* |  |
| Linking to other partners and resources as part of exit strategy | *Linking with local universities, volunteer students pursuing a Master’s in Clinical Psychology—who are required to complete around 500 hours of training to graduate—could fulfill these training hours under joint supervision by university professors and ICRC psychologists. There will be supervised by their professors, so in case one day we will not be able to fund this position and we will not have a psychosocial psychologist, we can contact them (professors) and the Sheikh could accept the master students to have their hours done and then they can provide the individual sessions and all of this is for free (or low cost of stipends etc). (ICRC)* |  |
| Financial barriers for continuation | *Honestly and in general, the local associations and communities, especially in our regions, have no sufficient means for sustainability. Without the support of foreign organizations, no one would have continued in this way. We have stopped working between (x) and the ICRC, and until now, many clinics are not covered. …It is impossible to rely on ourselves. (CAJA)* |  |
| ***Lessons for replication in other sites*** | |  |
| Knowing the dynamics and mandates | *You need to have the buy in by the health facility, then also to be in line with what is done in the country. Then you have to see who is allowed to do what, because also this plays a big role it a big shift, actually, in many in the Middle East, at least, from secondary care to primary care and the decentralization (ICRC).* |  |
| Expecting curveballs and the unexpected | *But like if it's in a place like Lebanon, you can have things coming up that you really would not expect biggest advice ….be prepared to anything because especially if I mean depends on the context, of course... (ICRC)* |  |
| The team will make or break the programme- engage inputs from staff on ground who will implement and sustain process | *An important thing is to learn the dynamics of the place you're gonna work. Because even if you're doing this kind of project, it's still the people there that are doing it. So, you need to really have a good team dynamic- So basically to to to to understand the team, the local teams’ dynamics, how they work. (ICRC)*  *(early on) have a bit more of involvement from the team itself (ICRC)*  *Study it first. Have certain strong process discuss it with the with the with the PHC staff, and the and get the trust. Get the feedback of the PHC staff, because the staff are the ones that are on the ground are the one who are doing the practice. So, try not to change a lot all at once, so really, do your homework. Take advice from the staff and then when you practice, definitely you will have gaps and things to change as you go along. (ICRC)* |  |
| Invest time and resources in team building, role clarity and work expectations | *from the beginning to put everyone together, helpful for services to be co-located, It is that people go together to some training, so to remove any hierarchy between, you know, a doctor or nurse, a physiotherapist and mental health specialist, …they are all together at the same level; Then also with practical exercises …realizing that you cannot ..not working together, you will not address the needs of the patients. Meaning it's it's really to to clarify roles and responsibilities and for from there also to identify uh task manager. (ICRC)* |  |
| Regular follow-ups on programme action points, training or capacity building case review and management | *From the beginning, also to have a few types of meetings- Team meetings where they discussed the organization of care and other meetings where they discuss about individual patient situations and involve patients. Also to get the feedback on what that service users and teams need beyond just medical care, but if there is anything else and find resources in the community (ICRC)* |  |
