# Supplementary material for: Integrating noncommunicable disease care in a public primary health care facility in North Lebanon: A qualitative study of implementation in a humanitarian crisis
Source: PLOS Glob Public Health. 2026 Apr 1;6(4):e0005518. doi: 10.1371/journal.pgph.0005518 (PMC13042628; doi:10.1371/journal.pgph.0005518)
Supplement: Attachment — Submitted filename: Resubmission letter - CAJA qualitative paper_21.07.25.docx [file pgph.0005518.s006.docx]

London School of Hygiene & Tropical Medicine

Keppel St

London WC1E 7HT

United Kingdom

21^st^ July 2025

Dear PLoS Global Public Health Editors,

Please find enclosed a Research Article submission for your consideration: **Integrating noncommunicable disease care in a public primary health care facility in North Lebanon: a qualitative study of implementation in a humanitarian crisis.**

This is a re-submission (original submission ID: PGPH-D-25-01531), as the submission system incorrectly listed the corresponding author as one of first authors, rather than senior author. We were asked to resubmit the manuscript and the issue has been rectified in the resubmission. The manuscript was not yet sent to reviewers.

We report the findings of a research study conducted with the ***International Committee of the Red Cross*** **(ICRC)** and their implementing partners in North Lebanon, the ***Chabab Al Ataa Al Jazeel Association (CAJA).*** We aimed to document the process of integrating primary health care services for people living with diabetes and/or hypertension (DM/HTN) within a primary health care in North Lebanon, to learn lessons and to inform implementation of similar programmes in other humanitarian settings.

Globally, the provision of care for noncommunicable diseases (NCDs) in humanitarian settings is increasing and implementing actors advocate for a primary-care-centred NCD model that strengthens the existing healthcare system. However, there are few documented examples of practical implementation of integrated NCD care within health systems in such settings. We previously published a [Theory of Change](https://conflictandhealth.biomedcentral.com/articles/10.1186/s13031-023-00532-x) (ToC) around the integration of these services, co-produced with the ICRC and the CAJA team. The current study reflects on the ToC process, identifies key facilitators and remaining gaps around implementation, and makes concrete recommendations for integrating NCD care in humanitarian settings.

Drawing on the perspectives of patients, healthcare providers, and stakeholders, and using a conceptual framework, our qualitative study examines the integration of co-located mental health and psychosocial support, rehabilitation and primary care for people living with NCDs attending the CAJA Primary Care Centre in Bireh, Aakar Governorate, North Lebanon.

This is a re-submission (original submission ID: PGPH-D-25-01531), as the submission system incorrectly listed the corresponding author as one of first authors, rather than senior author.

We hope that you will consider this manuscript for publication.

We look forward to your response.

Yours sincerely,

Éimhín Ansbro, on behalf of all co-authors
