# Supplementary material for: Integrating noncommunicable disease care in a public primary health care facility in North Lebanon: A qualitative study of implementation in a humanitarian crisis
Source: PLOS Glob Public Health. 2026 Apr 1;6(4):e0005518. doi: 10.1371/journal.pgph.0005518 (PMC13042628; doi:10.1371/journal.pgph.0005518)
Supplement: Attachment — Submitted filename: Response to reviewers 18.11.25.docx [file pgph.0005518.s007.docx]

September 18, 2025

Dear Dr. Bhandari,

We are pleased to resubmit the revised version of Manuscript entitled: “Integrating noncommunicable disease care in a public primary health care facility in North Lebanon: a qualitative study of implementation in a humanitarian crisis”

Ref: Submission ID: PGPH-D-25-02036

We would like to thank the editor and the reviewers for their constructive and insightful comments, which we have carefully addressed. All responses to the reviewers’ comments are provided in italics and prefaced by “Author response.” Modifications in the manuscript are highlighted in the “tracked changes” version in yellow. Please find below a summary of how we addressed the comments.

We have now additionally responded to editorial requests regarding Table placement and Figure files.

My co-authors and I sincerely appreciate the opportunity to resubmit and are enthusiastic about the possibility of publication.

Dr Éimhín Ansbro,

London School of Hygiene & Tropical Medicine, on behalf of all co-authors.

______________________________________________________________________________

**Reviewer #1:** Good overview of interventions that can be done to improve chronic disease prevention and management. Could also discuss treatments and whether there was any decrease in hospitalizations related to chronic disease as well.

***Author’s reply: We appreciate the reviewer’s valuable comment. This qualitative study, conducted alongside the first year of programme implementation, focused on identifying lessons learned and real-time adaptations to support the model’s long-term sustainability. Data on hospitalizations are inherently difficult to obtain in this context; however, a follow-up study is underway to assess treatment components and their effects on patients, which will provide further evidence on the effectiveness of the interventions. The results of this evaluation will be presented in a subsequent paper.***

Reviewer #2: These notes provide structured feedback based on key domains of qualitative research appraisal (drawing from established qualitative research evaluation frameworks). The comments are intended to guide authors in strengthening methodological clarity, transparency, and reporting consistency.

Study Aim and Design
• Research question clearly stated and suitable for qualitative inquiry: Yes
• Qualitative design appropriate to address the question: Yes
• Rationale for using a qualitative approach: Yes

Sampling and Participants
• Sampling strategy description (e.g., purposive, theoretical):

Line 240–244: Authors report CAJA service users were purposefully sampled with information on the inclusion criteria and how participants were recruited. Consider providing additional information on the specific type of purposive sampling strategy and rationale for selecting this method.

***Author’s reply:*** ***We thank the reviewer for this comment. We have clarified the purposive sampling strategy and rationale in the revised manuscript. Specifically, we now indicate that maximum variation purposive sampling was used to select eight CAJA service users, allowing us to capture diverse perspectives and experiences. Modifications have been made in the Methods section to improve transparency and provide a clear rationale for this approach (Line 241-249).***

• Participant characteristics and recruitment methods appropriate and well-described: Yes

• Sample size adequate for achieving data saturation:
Line 249: The authors mention that data saturation was achieved, which supports adequacy of the dataset. However, the total number of participants (or interviews) is not reported. Including this information would improve transparency and allow readers to assess the depth and scope of data collection.

***Author’s reply: We thank the reviewer for this suggestion. We have now reported the total number of participants: 20 in total, including eight CAJA facility staff, four ICRC staff (three Lebanon-based and one Geneva-based), and eight CAJA service users with type 1 or type 2 diabetes and/or hypertension (Line 220). Data collection continued until data saturation was reached, supporting the adequacy of the dataset.***

Data Collection

• Data collection methods clearly described: Yes
• Setting and context of data collection specified: Yes
• Reflexivity (researcher’s role, assumptions, influence): Yes

Data Analysis
• Analytic approach described and justified (e.g., thematic, grounded theory): Yes
• Coding and theme development steps transparent: Yes
• Findings supported by illustrative quotes or data extracts: Yes

Trustworthiness and Rigor
• Credibility (triangulation, member checking):
The manuscript indicates triangulation was used. Detailing how member checking was implemented would further strengthen credibility.

***Author’s reply: We thank the reviewer for this valuable comment. We have now clarified the approach to member checking in the manuscript. Specifically, we added details in Section 2.5 to indicate that regular team discussions were held with LSHTM and ICRC colleagues to support reflexivity, ensure analytic rigour, and facilitate organic member checking among staff participants. These iterative meetings contributed to consensus building and refinement of emerging themes. In addition, we expanded Section 2.7 (Patient and Public Involvement) to explain that while member checking with service users had been planned, it could not be implemented due to time constraints and contextual challenges. However, several staff participants were engaged throughout the analysis and manuscript development process, serving as an indirect form of member checking consistent with participatory research approaches.***

• Dependability (audit trail, documentation):

Dependability appears supported through clear analytic documentation and mention of an audit trail. Additional description of coding logs or decision trails could enhance this section.

***Author’s reply: Coding and analysis were conducted manually (using MS Excel and Word) by LV (LSHTM). Regular team discussions were held with LSHTM and ICRC colleagues to support reflexivity, ensure analytic rigour, organic member checking among staff participants, and enhance the reliability and internal validity of interpretations. These iterative analysis meetings contributed to consensus building and refinement of emerging themes (pg 15, section 2.5), and axial codes are provided in the supplement.***

• Reflexivity or confirmability: Yes
• Transferability (contextual detail for generalization): Providing rich description of the study context could assist readers in assessing transferability.
***Author’s reply: The manuscript already provides extensive contextual detail to support transferability. Specifically, three paragraphs in the Background section (pages 5–6) describe the broader contextual setting, including the operational and humanitarian nature of the CAJA Primary Health Care (PHC) program and its integration within the public PHC network. Further description of the study context is also presented in the Methods section (page 9).***

***To enhance clarity for readers, we have now added a linking sentence in the manuscript that re-mentions the operational nature, timing, and circumstances of the program “as described in the background section.” Additionally, contextual factors are further reflected and discussed in the Results section, which collectively strengthens the study’s transferability.***

Reporting and Interpretation
• Findings clearly presented and logically organized: Yes
• Findings linked to existing literature or theory: Yes
• Participant voices presented authentically: Yes

Ethical Considerations
• Ethical approval obtained and stated: Yes
• Informed consent described: Yes
• Confidentiality and participant protection addressed:
***Author’s reply: We thank the reviewer for this comment. We have clarified in the Methods section that participants were assigned unique codes, personal identifiers were removed from transcripts, quotes used in the manuscript were anonymized, and all data were stored securely with access restricted to the research team to ensure de-identification and confidentiality.***

Overall Assessment

• Paper clearly written and logically structured: Yes
• Conclusions align with data presented: Yes
• Limitations and implications for practice or research discussed: Yes

Overall, this is a well-organized and thoughtfully executed qualitative study. Enhancing reporting around sampling rationale, participant numbers, and the processes underpinning credibility and dependability would further strengthen methodological transparency.

 Figure Resubmissions:

While revising your submission, we strongly recommend that you use PLOS’s NAAS tool (<https://ngplosjournals.pagemajik.ai/artanalysis>) to test your figure files. NAAS can convert your figure files to the TIFF file type and meet basic requirements (such as print size, resolution), or provide you with a report on issues that do not meet our requirements and that NAAS cannot fix.

After uploading your figures to PLOS’s NAAS tool - <https://ngplosjournals.pagemajik.ai/artanalysis>, NAAS will process the files provided and display the results in the "Uploaded Files" section of the page as the processing is complete. If the uploaded figures meet our requirements (or NAAS is able to fix the files to meet our requirements), the figure will be marked as "fixed" above. If NAAS is unable to fix the files, a red "failed" label will appear above. When NAAS has confirmed that the figure files meet our requirements, please download the file via the download option, and include these NAAS processed figure files when submitting your revised manuscript.
